# Oral Supplements of Combined *Bacillus licheniformis* Zhengchangsheng® and Xylooligosaccharides Improve High-Fat Diet-Induced Obesity and Modulate the Gut Microbiota in Rats

**DOI:** 10.1155/2020/9067821

**Published:** 2020-05-18

**Authors:** Yuyuan Li, Man Liu, He Liu, Xiaoqing Wei, Xianying Su, Ming Li, Jieli Yuan

**Affiliations:** ^1^Advanced Institute for Medical Sciences, Dalian Medical University, Dalian, China; ^2^Department of Microecology, College of Basic Medical Science, Dalian Medical University, Dalian, China; ^3^The Core Laboratory of Medical Molecular Biology of Liaoning Province, Dalian Medical University, Dalian, China; ^4^Research Institute of Northeastern Pharmaceutical Group (NEPG), Shenyang, China

## Abstract

Gut dysbiosis induced by high-fat diet (HFD) may result in low-grade inflammation leading to diverse inflammatory diseases. The beneficial effects of probiotics and prebiotics on obesity have been reported previously. However, their benefits in promoting human health and the underlying mechanisms still need to be further characterized. This study is aimed at understanding how probiotic *Bacillus licheniformis* Zhengchangsheng® (BL) and prebiotic xylooligosaccharides (XOS) influence the health of a rat model with HF (60 kcal %) diet-induced obesity. Five groups of male Sprague Dawley (SD) rats were fed a normal fat diet (CON) or an HFD with or without BL and XOS supplementation for 3 weeks. Lipid profiles, inflammatory biomarkers, and microbiota composition were analyzed at the end of the experiment. Rats fed an HFD exhibited increased body weight and disordered lipid metabolism. In contrast, combined BL and XOS supplementation inhibited body weight gain and returned lipid metabolism to normal. Furthermore, BL and XOS administration changed the gut microbiota composition and modulated specific bacteria such as *Prevotellaceae*, *Desulfovibrionaceae*, and *Ruminococcaceae*. In addition, supplements of combined BL and XOS obviously reduced the serum LPS level, which was significantly related to microbial variations. Our findings suggest that modulation of the gut microbiota as a result of probiotic BL and prebiotic XOS supplementation has a positive effect on HFD-induced obesity in rats.

## 1. Introduction

Obesity is a global epidemic; the incidence of which is 10.7% in China, 12.8% in the European Union, and 30.4% in the USA [1–3]. In 2035, 39% of people in today's world will be affected by obesity according to the World Health Organization (WHO) [4]. Epidemiologic studies have shown that obesity imposes a heavy burden on national health care systems because it is tightly associated with chronic illnesses, including type II diabetes, cardiovascular disease, cancer, and fatty liver disease [5]. The development of obesity is complex and involves both genetic and environmental factors. There is a growing evidence to show that alteration of gut microbiota composition may induce low-grade inflammation and is identified as an important element in close association with the obesity and obesity-induced metabolic disorders [6–9]. Microbiota-induced low-grade inflammation is mainly induced by lipopolysaccharides (LPS), increased plasma levels of which are sufficient to trigger obesity [10, 11]. Two factors may contribute to the increased entry of LPS into the body, resulting in metabolic endotoxemia and eventually obesity. One is the imbalance in the composition of the gut microbiota, which increases the LPS-bearing bacteria population, directly elevating the gut levels of LPS. The other is the impairment of the gut barrier function, which allows LPS to more easily enter the circulatory systems [10]. Moreover, the reduced *Firmicutes*/*Bacteroidetes* ratio has been associated with improved glucose levels, body weight, and fat reduction [12, 13]. Thus, the gut microbiota represents a potential therapeutic target for the development of drugs or nutritional interventions for obesity.

A number of studies have demonstrated that selective modulation of gut microbiota using probiotics and/or prebiotics has emerged as a potential therapy for the treatment of obesity [14, 15]. According to the Food and Agriculture Organization of the United Nations (FAO) and WHO, probiotics are defined as “live microorganisms which, when administered in adequate amounts, confer a health benefit on the host” [16]. The probiotic *Bacillus licheniformis* Zhengchangsheng® (BL), which is “generally recognized as safe,” has been used extensively as an antiviral and immunoregulatory agent in clinical treatment [17–19]. In our previous study, we demonstrate that BL can attenuate dextran sulphate sodium (DSS)-induced colitis and modulate dysbiosis during inflammatory bowel disease (IBD) [20]. Prebiotics have been defined by FAO/WHO as “non-digestible food ingredients that beneficially affect the host by selectively stimulating the growth and/or activity of one or a limited number of bacterial species already established in the colon, and thus improve the host health” [21]. The putative prebiotic xylooligosaccharides (XOS) are sugar oligomers made up of xylose units, which were found to increase the fecal *Bifidobacterium* populations [22], and also have the potential to improve the management of blood sugars and cholesterol [23, 24].

In this study, we further investigated whether the consumption of BL and XOS combination could ameliorate high-fat diet- (HFD-) induced obesity in rats through the manipulation of gut microbiota dysbiosis and reduction of systematic inflammation.

## 2. Materials and Methods

### 2.1. Animal and Dietary Intervention

Ten-week-old male Sprague Dawley (SD) rats weighing 380-420 g (*n* = 30) were obtained from the Experimental Animal House of Dalian Medical University, Dalian, China. All rats were kept in an air-conditioned room with 12 h light and dark cycle. They were fasted for 24 h prior to all experiments. The animal experiments were conducted with the approval of the Animal Research Committee of Dalian Medical University in accordance with the laboratory's animal ethics guidelines (SYXK (Liao) 2014-0002).

After one week of adaptation on chow diet, rats were randomly divided into five groups (*n* = 6) and fed on standard chow or high fat: normal fat diet (CON), high-fat diet (HF), and HFD-fed rats that were treated with 0.4 ml XOS at 2 g/ml dissolved in PBS (XOS), Zhengchangsheng strain (CMCC63516, isolated and cultured by the Northeast Pharmaceutical Group, Shenyang No.1 Pharmaceutical Co. Ltd. China, Lot S10950019) at 7.5 × 10^8^ CFU/ml suspended in PBS (BL), or the combination of XOS and BL (XOS-BL) groups. Mice in the CON group were administrated a normal diet; the other groups were fed an HFD (60% fat, 20% protein, 20% carbohydrate, as a percentage of total kcal, H10060, Beijing HFK Bioscience Co., Ltd.). The XOS, BL, and XOS-BL groups were gavaged with 0.4 ml of XOS, BL, and XOS-BL once a day throughout three weeks. The CON and HF groups were instead gavaged with 0.4 ml PBS. Body weight gain and other parameters were evaluated once a week. Feces were collected at the end of 3 wk for 16S rRNA sequencing. After 3 wk, the rats were euthanized for blood and tissue collection. The epididymal fat and perirenal fat were weighed, and the ratios of epididymal fat and perirenal fat to body weight were calculated.

### 2.2. Biochemical Analysis

Blood samples were collected from the heart and separated to the plasma at 3,000 rpm for 15 min at 25°C and then stored at -80°C for use. Liver tissues were mixed with saline at a ratio of 1 : 9 and homogenized. The mixture was centrifuged at 12,000 rpm for 10 min at 4°C, and then the supernatant was retained for subsequent analysis. The concentrations of serum total cholesterols (TC), triglycerides (TG), high-density lipoprotein cholesterol (HDL-C), and low-density lipoprotein cholesterol (LDL-C) were detected by enzymatic methods according to the instructions of relevant assay kits (Nanjing Jiancheng Institute of Biotechnology, Nanjing, China). The serum and liver levels of LPS were measured with ELISA kits (ShangHai Lengton Bioscience Co., LTD, Shanghai, China).

### 2.3. Denaturing Gradient Gel Electrophoresis (DGGE)

The fecal DNA was extracted using the QIAamp DNA Stool Mini Kit (Qiagen, Hilden, Germany). PCR amplification was conducted using the universal bacterial primers F338+GC clamp and R518, which targets the hypervariable V3 region of 16S rRNA gene. The resulting 16S rDNA amplicons were analyzed by DGGE fingerprinting analysis using the DCode system from Bio-Rad (Hercules, CA, USA) according to descriptions of Joossens et al. [25]. Next, the DGGE images were analyzed with Quantity One image analysis software (version 4.6.1; Bio-Rad). Similarities were displayed graphically as a dendrogram.

### 2.4. Illumina HiSeq Sequencing and Bioinformatics Analysis

The universal primers (520F, 802R) were used to amplify the V3-V4 region of 16S ribosomal DNA from metagenomic DNA in rat feces of five groups (*n* = 5‐6 for each group). PCR products were checked by 1.5% (*w*/*v*) agarose gel electrophoresis in 0.5 mg/ml ethidium bromide and purified. The sequencing was performed on Illumina HiSeq platform by Novogene (Beijing, China) [26]. Operational taxonomic units (OTUs) present in 50% or more of the fecal samples were identified as core OTUs. Based on the results of OTUs, alpha and beta diversities were analyzed subsequently. Alpha diversity was evaluated by observed species and Shannon index. Community richness was evaluated by Chao1 index. Beta diversity was evaluated by Principal Coordinate Analysis (PCoA) based on the weighted UniFrac analysis and Unweighted Pair-Group Method with Arithmetic mean clustering (UPGMA). Linear Discriminant Analysis Effect Size (LEfSe) was used to identify the bacterial taxa differentially represented between groups at different taxonomic levels. A Linear Discriminant Analysis (LDA) was used to estimate the effect size of each differentially abundant feature.

### 2.5. Statistical Analysis

All the experiments were performed at least two times in triplicates, and data were presented as arithmetic mean ± standard error of mean (SEM). The data sets involved in more than two groups were assessed by one-way ANOVA followed by Tukey's test using GraphPad Prism (version 7.04; GraphPad Software Inc., La Jolla, CA, USA). The significance for PCoA (beta diversity) analyses, which was tested with multivariate permutation tests using the nonparametric method “Adonis” included in the package “vegan” of the QIIME-incorporated version of “R”. Community comparison was evaluated using Student's *t* test. A *P* value of less than 0.05 was considered statistically significant.

## 3. Results

### 3.1. Effects of BL Combined XOS on Body Weight, Lipid Parameters, and Inflammatory Biomarkers of HFD-Fed Rats

**Figure 1:**
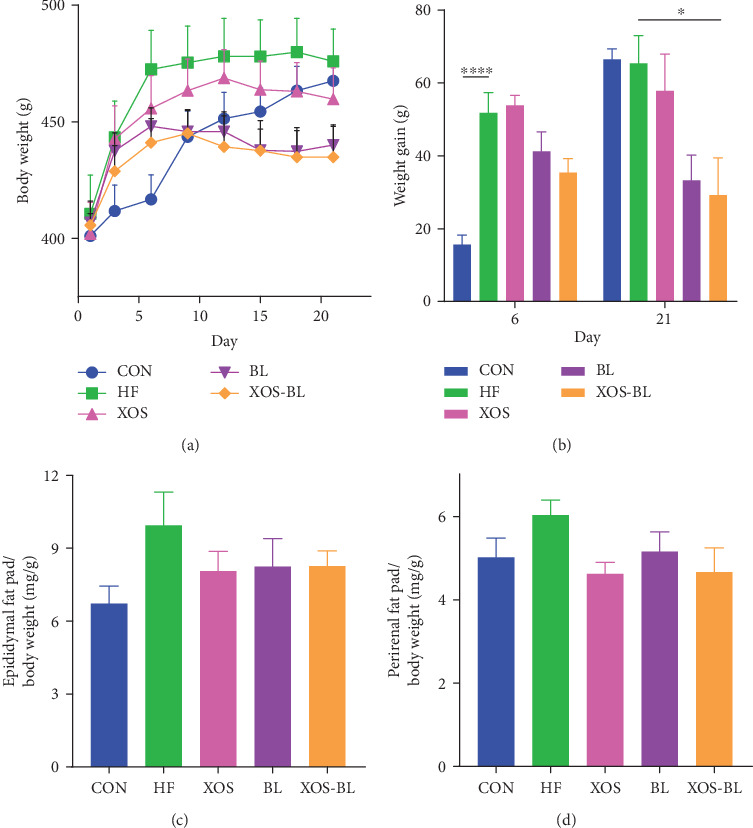
Effects of *Bacillus licheniformis* (BL) and xylooligosaccharides (XOS) on body composition. (a) Total body weight; (b) total weight gain on days 6 and 21; (c) epididymal and (d) perirenal fat pad weights were normalized against total body weight. The differences were assessed by one-way ANOVA with Tukey's test. ^∗^*P* < 0.05; data represent mean ± SEM of six mice in each group.

**Figure 2:**
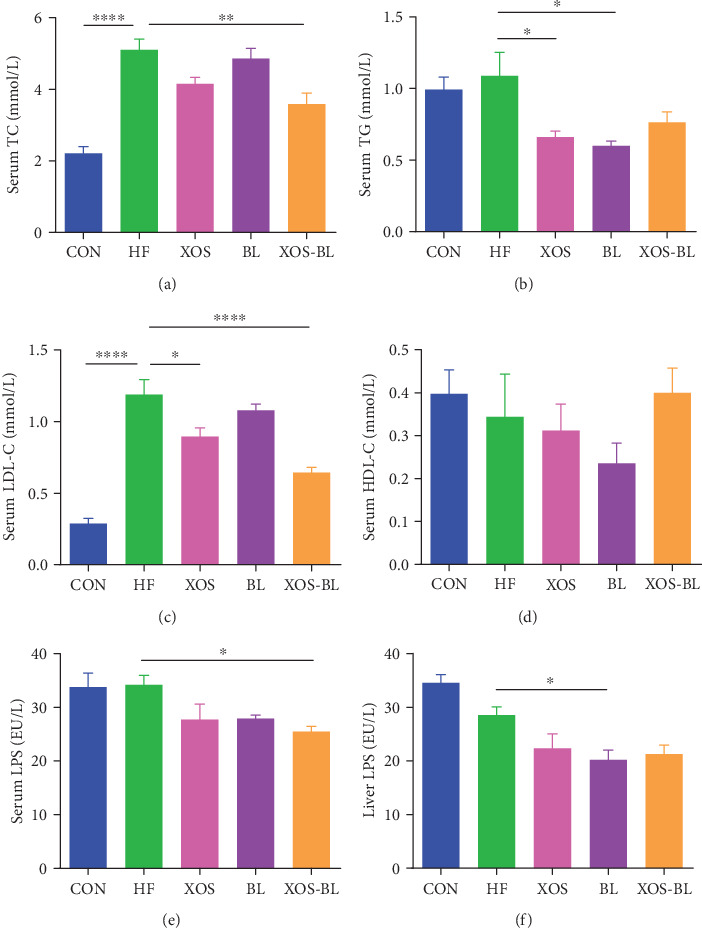
Effects of BL and XOS on serum lipid profile and serum and liver lipopolysaccharides. (a) Serum total cholesterol (TC), (b) serum triglyceride (TG), (c) serum low-density lipoprotein cholesterol (LDL-C), (d) serum high-density lipoprotein cholesterol (HDL-C), (e) serum lipopolysaccharides (LPS), and (f) liver LPS levels of rats from different experimental groups were analyzed by ELISA. The differences were assessed by one-way ANOVA with Tukey's test. ^∗^*P* < 0.05, ^∗∗^*P* < 0.01, and ^∗∗∗∗^*P* < 0.0001; data represent mean ± SEM of six mice in each group.

To investigate the effect of the combination of BL and XOS on obesity, we established the obese rat model by feeding the male SD rats a high-fat diet with 60% of fat content. The body weights of the rats and the excised tissue content were recorded. Our results showed that HFD had induced significant increases in final body weight and body weight gain after day 6 compared with the control group (*P* < 0.0001; Figures 1(a) and 1(b)). Combined BL and XOS supplementation (XOS-BL) reduced this increase tendentiously in HFD-fed rats (*P* = 0.0588; Figure 1(b)). After 3 weeks, combined BL and XOS supplementation significantly reduced body weight gain in HFD-fed rats (*P* = 0.0323; Figure 1(b)). However, there was no significant difference among the groups in the total weight of the epididymal and perirenal fat pads (Figures 1(c) and 1(d)). The levels of serum TC and LDL-C were significantly decreased in the XOS-BL group compared with the HF group (*P* = 0.0027 and *P* < 0.0001, respectively; Figures 2(a) and 2(c)). The TG level in the XOS-BL group was tendentiously decreased, while the HDL-C level was tendentiously increased compared with the HF group (Figures 2(b) and 2(d)). Moreover, we also evaluated anti-inflammatory ability of BL and XOS in obese rats. We observed a significant decrease in serum LPS level in the XOS-BL group compared with the HF group, implying the alleviation of endotoxemia and systemic chronic inflammation (*P* = 0.0346; Figure 2(e)). Rat liver was also evaluated for the levels of LPS after treatment of BL and/or XOS. The liver LPS was significantly lower in the BL group compared to the HF group (*P* = 0.049; Figure 2(f)). These results illustrated that combined BL and prebiotic XOS supplementation could effectively ameliorate the body weight and serum parameters of the obese rats.

### 3.2. Effect of BL Combined XOS on Gut Microbiota of HFD-Fed Rats

**Figure 3:**
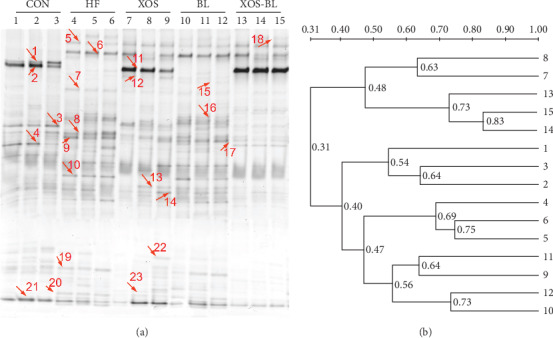
Preliminary evaluation of the intestinal microbiota in rats by PCR-denaturing gradient gel electrophoresis (DGGE). (a) DGGE profiles of the V3 region of 16S rRNA gene amplicons derived from fecal DNA of rats in each group. PCR was performed using a F338-GC/R518 set of primers. Lanes indicate the microbial groups in fecal samples from different experimental groups (*n* = 3) taken at day 21; (b) cluster analysis of the DGGE profiles. The dendrogram was constructed using the UPGMA method.

The intestinal microbiota of rats was analyzed by PCR-DGGE using universal primers of the bacterial 16S rDNA-V3 region (Figure 3(a)). The number of bands in every DGGE profile was determined as the diversity of the intestinal microbiota. Our data showed that the abundance of intestinal microbes was significantly different between the control group and the HF group of rats. The dendrogram constructed based on DGGE profile supported that they joined in different clusters.  Figure 3(b) displays that the HF and the BL groups joined together, and the XOS and XOS-BL groups joined in one cluster. In Figure 3(a), 23 bands that were obviously different among groups were selected and excised for sequencing analysis. To verify the resolution capability of DGGE, bands in the same position but in different lanes were cut and sequenced. Sequencing results demonstrated that they belong to the same bacterial groups and are summarized in Table 1. The microbiota of the CON group is characterized by the presence of genera *Lactobacillus* (bands 1 and 3). The bands corresponding to *Phascolarctobacterium succinatutens* (band 5) and *Bacteroides uniformis* (bands 6 and 7) were identified in groups administrated by high-fat diet. The XOS and XOS-BL groups exhibited intense bands corresponding to *P. succinatutens*. The bands corresponding to *Prevotella copri* (bands 2 and 11) were detected in the CON, XOS, and XOS-BL groups, respectively. The pattern of the XOS and BL groups exhibits the bands corresponding to *Parabacteroides distasonis*. In particular, band 23 corresponding to *Bifidobacterium pseudolongum* was identified in the XOS group.

**Figure 4:**
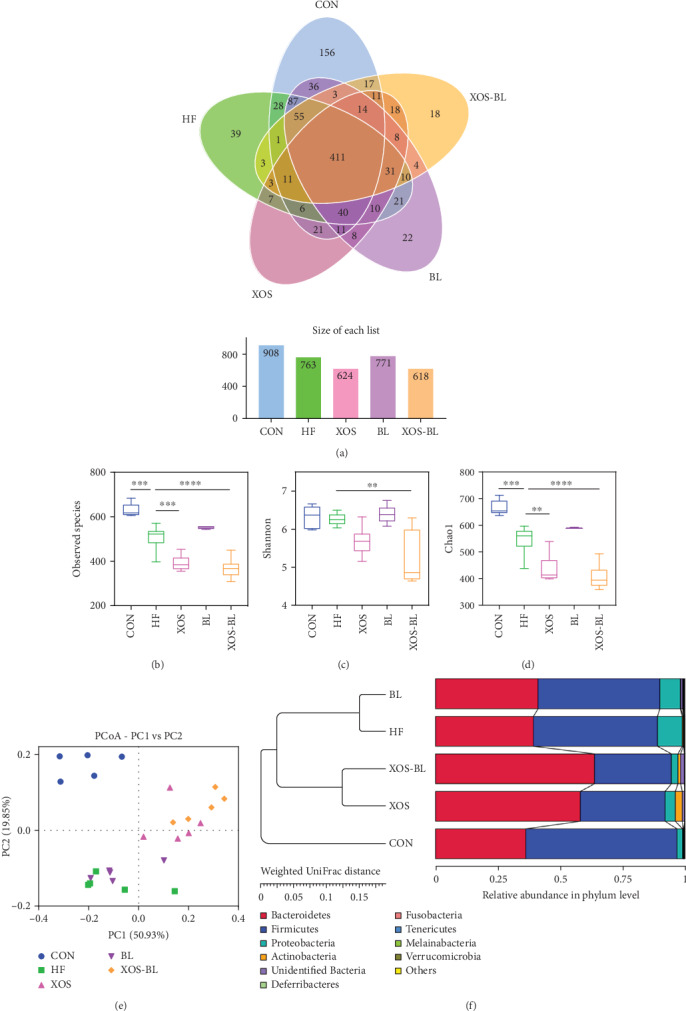
Evaluation of Illumina HiSeq sequencing data showing that BL and XOS could modulate the overall structure of gut microbiota in HFD-fed rats. (a) Venn diagram of shared and independent bacterial OTUs in different experimental groups (*n* = 5‐6); (b) comparison of the observed species of different groups; (c) comparison of the Shannon index of different groups; (d) comparison of the Chao1 index of different groups; (e) principal coordinate analysis (PCoA) based on weighted UniFrac distances among different samples. PC1 and PC2 account for 70.78% of the variation; (f) multivariate analysis of variance from PCoA matrix scores using the UPGMA method based on weighted UniFrac distances. ^∗∗^*P* < 0.01; ^∗∗∗^*P* < 0.001; ^∗∗∗∗^*P* < 0.0001.

To characterize the changing of gut microbiota systematically in HFD-fed rats administrated with BL and XOS, we performed metagenomic analysis of the V3-V4 region of 16S rRNA gene sequences. After subsampling, 1,124 operational taxonomic units (OTUs) were classified at 97% similarity (Figure 4). The OTU number of the control group was 908, which was much higher than the other groups. Among them, 156 OTUs were found unique to the control group, 39 OTUs were unique to the HF group, and 14 OTUs, 22 OTUs, and 18 OTUs were found unique to the XOS group, the BL group, and the XOS-BL group, respectively (Figure 4(a)). Alpha diversity analysis showed that obvious differences could be observed between the groups (Figures 4(b)–4(d)), suggesting that the diversity and richness of the gut microbiota were significantly altered by HFD and administration of BL and XOS. Then, the similarities and differences between the gut microbiota communities of groups were analyzed by PCoA and UPGMA (Figures 4(e) and 4(f)). It revealed a distinct clustering of the gut microbiota composition for each group (control vs. HF, *R* = 0.615, *P* = 0.001; HF vs. XOS, *R* = 0.441, *P* = 0.001; HF vs. BL, *R* = 0.075, *P* = 0.606; HF vs. XOS-BL, *R* = 0.454, *P* = 0.001), indicating that the intake pattern of XOS-BL caused certain influence on the gut microbiota composition of the HFD-fed rats.

**Figure 5:**
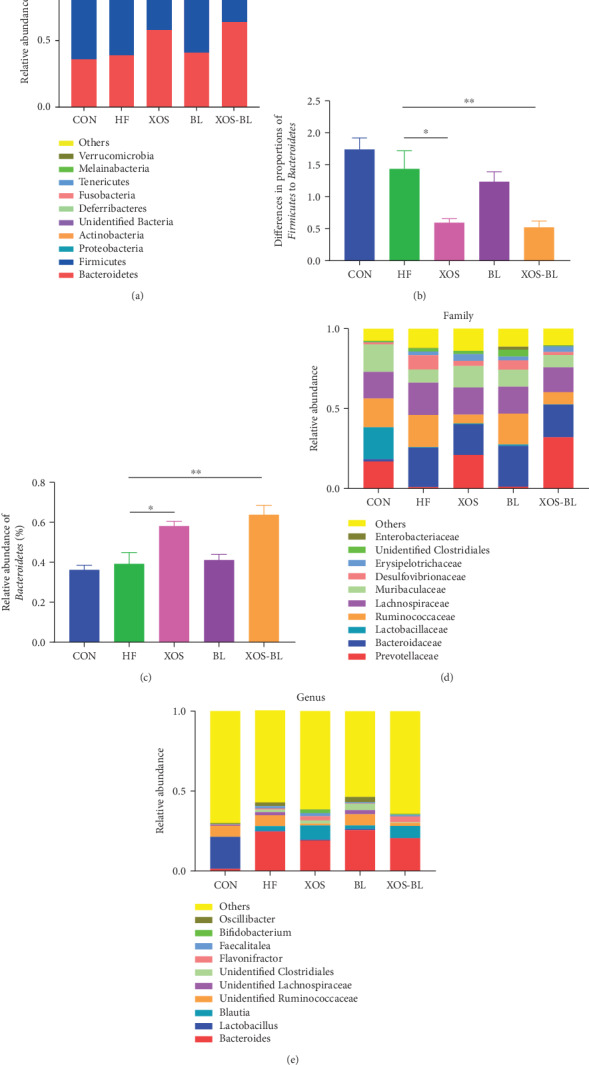
Composition analysis of gut microbiota at different taxonomic levels among all samples. (a) The average abundance of microbial community in different groups at phylum level; (b) the ratio of *Firmicutes* to *Bacteroidetes*; (c) proportion of *Bacteroidetes*; (d) microbial community bar plot at family level; (e) microbial community bar plot at genus level. All values are mean ± SEM (*n* = 5‐6). The differences were assessed by one-way ANOVA with Tukey's test. ^∗^*P* < 0.05; ^∗∗^*P* < 0.01.

**Figure 6:**
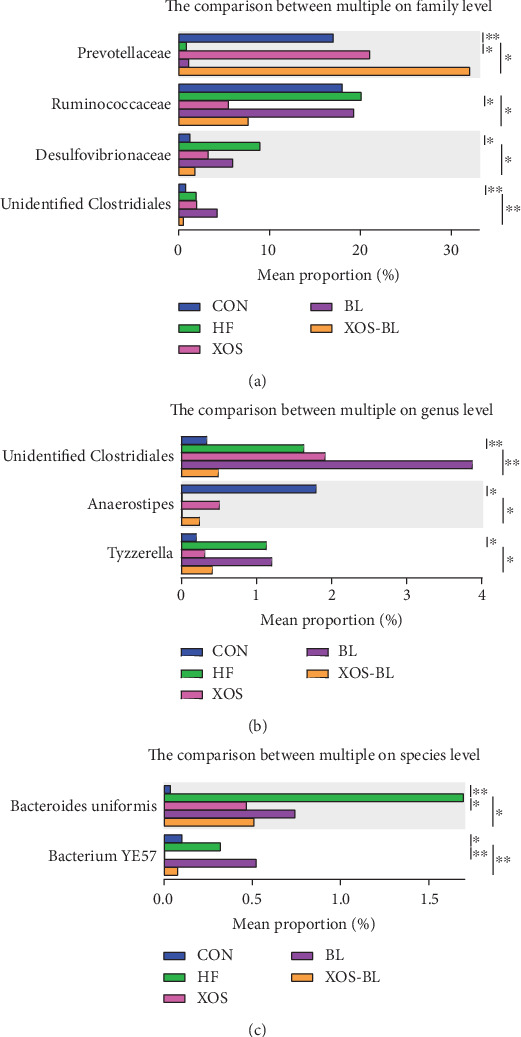
The analysis of species difference in different groups. The comparison between HF and the other four groups in family (a), genus (b), and species (c) level based on *t* test. ^∗^*P* < 0.05; ^∗∗^*P* < 0.01.

The taxonomic analysis suggested that the fecal microbiota of SD rats used in this study was represented by three major phyla: *Bacteroidetes*, *Firmicutes*, and *Proteobacteria* (Figure 5(a)). The ratio of *Firmicutes* to *Bacteroidetes* (F/B), a widely used marker of gut dysbiosis [27], was found significantly lower in the XOS-BL group when compared to the HF group (*P* < 0.01; Figure 5(b)). Our results showed that the main taxons that contributed to the changes in *Firmicutes* and *Bacteroidetes* were genera *Bacteroides* and *Lactobacillus*. In comparison to the HFD group, mice of the CON group had significantly more abundance in total *Lactobacillus* spp. (+19.7%, *P* < 0.01). The families of *Prevotellaceae* and *Bacteroides* caccae were differentially abundant in the XOS-BL group (*P* < 0.01; Figure 5(c)). The SCFA-producing genera *Parabacteroides* [28, 29] (members of Bacteroidetes) and genera of *Blautia* [30] (members of *Firmicutes*) were key taxons in mice of the XOS group (Figures 5(d) and 5(e)). The statistical differences at the family, genus, and species levels were further tested. At the family level, *Prevotellaceae* was elevated dramatically, while *Ruminococcaceae*, *Desulfovibrionaceae,* and *unidentified Clostridiales* were significantly reduced by XOS and BL supplementation (Figure 6(a)). At the genus level, three representative genera were altered by XOS and BL treatment. *Anaerostipes* was significantly increased after XOS and BL treatment, while *unidentified Clostridiales* and *Tyzzerella* were significantly decreased after XOS and BL treatment (Figure 6(b). At the species level, we found that 2 species were altered by XOS and BL treatment. *Bacteroides uniformis* and *bacterium* YE57 were significantly reduced by intervention of XOS combined with BL (Figure 6(c)).

### 3.3. Biomarkers in Each Group

**Figure 7:**
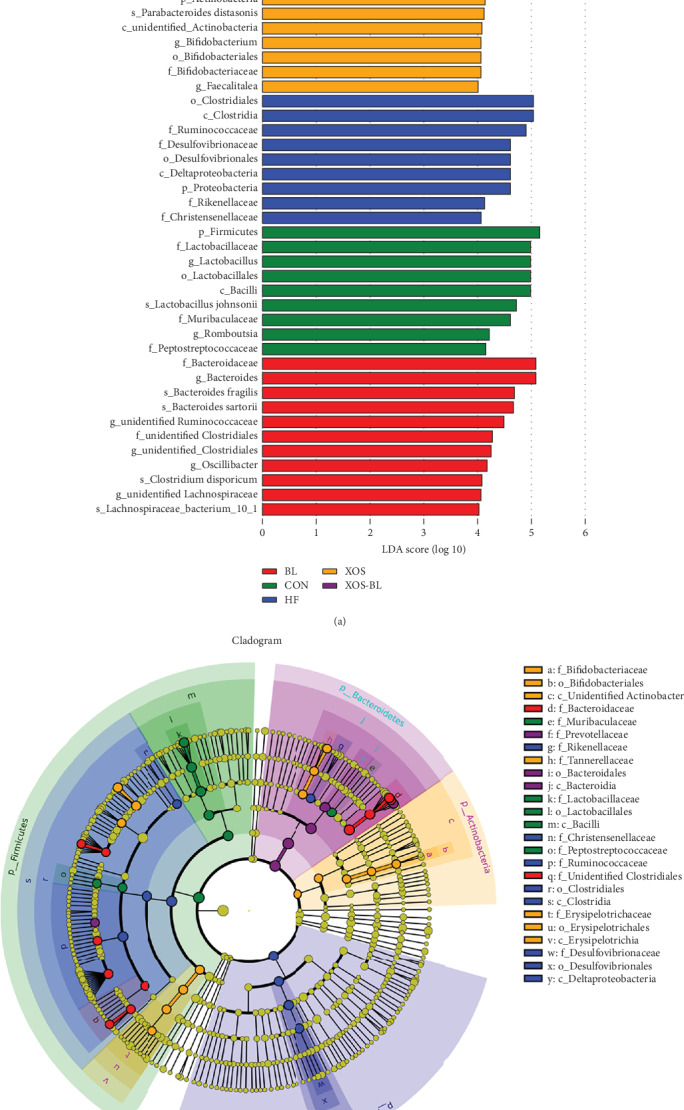
LEfSe analysis of intestinal microbiota among different groups. (a) LEfSe identified the most differentially abundant bacterial taxons among groups. Group-specific enriched taxa are indicated with a positive LDA score bar with different colors. Only taxa meeting an LDA significant threshold > 4 are shown; (b) taxonomic cladogram obtained from LEfSe analysis of 16S rDNA sequences. The brightness of each dot is proportional to its effect size.

The metagenomic analysis LEfSe approach was used to identify the key phylotypes responsible for the differences among the groups. The LDA scoring plot displayed the dominant bacteria (LDA>4) in each group. *Lactobacillaceae* was the most abundant in the control rats, while *Desulfovibrionaceae*, *Rikenellaceae*, and *Ruminococcaceae* were most abundant in the HFD-fed rats. *Bifidobacteriaceae* and *Erysipelotrichaceae* were most abundant in the XOS-treated rats; *Bacteroidaceae* was the most abundant in BL-treated rats; and *Prevotellaceae* was the most abundant in the XOS+BL-treated rats. These bacteria were the major phylotypes that contributed to the differences between the intestinal microbiota of different groups (Figure 7). Moreover, this result is consistent with that of DGGE in Figure 3 and Table 1.

### 3.4. Associations between LPS Levels and Microbial Taxa

**Figure 8:**
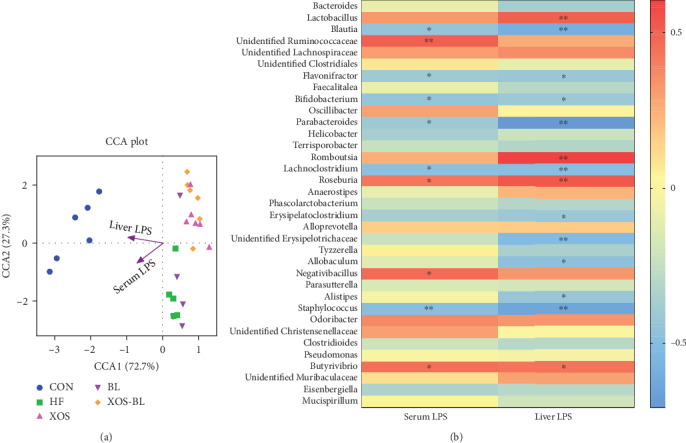
The correlation between intestinal bacterial groups and LPS levels in serum and liver. (a) Canonical correlation analysis (CCA) analysis. Arrows represent the variables serum LPS and liver LPS and indicate the direction and magnitude of the variables associated with bacterial community structure; (b) the correlation between the relative abundance of different microbial groups at genus level and the levels of LPS in the serum and liver was tested by Spearman's correlation method. The positive correlation was displayed as correlation value > 0, and the negative correlation was displayed as correlation value < 0; statistically significant correlation was displayed as ^∗^*P* < 0.05 and ^∗∗^*P* < 0.01.

Mantel tests and the canonical correlation analysis (CCA) indicated that the serum and liver levels of LPS were the major factors contributing to the differences between the bacterial communities and environmental factors (*P* = 0.005, Figure 8(a)). And the correlation between gut microbiota composition and LPS levels was also assessed by Spearman's algorithm in the present research (Figure 8(b)). The abundance of some of the gut microbes, including *Lactobacillus*, unidentified *Ruminococcaceae*, *Roseburia*, and *Butyrivibrio*, showed a positive relationship with serum and liver LPS levels; by contrast, the abundance of *Blautia*, *Flavonifractor*, *Bifidobacterium*, *Parabacteroides*, *Lachnoclostridium*, *Erysipelatoclostridium*, and *Staphylococcus* were negatively correlated with serum and liver LPS levels. As LPS are typically produced by gram-negative bacteria, *Lactobacillus*, *Roseburia*, and *Butyrivibrio* are gram-positive bacteria and are broadly reported to possess LPS-decreasing activity. But the present results showed a positive relationship to LPS. This may be due to the small group of analysis data.

## 4. Discussion

Extensive evidence indicates that the dysbiosis of gut microbiota is related to the pathogenesis of obesity and associated metabolic diseases [17]. Recently, the effects of dietary manipulation with probiotics and prebiotics on metabolic disorders have been studied [31]. In the current study, we used an HFD-induced model to investigate the effects of probiotic BL and prebiotic XOS on the gut microbiota of obese rats. Our results suggested that supplements of combined BL and XOS ameliorated negative effects of obesity, including weight gain, and elevated serum TC, LDL-C, and LPS levels. Moreover, BL and XOS administration caused a significant change in the gut microbiota by increasing the proportion of *Bacteroidetes* at the phylum level as well as its family *Prevotellaceae* and decreasing the F/B ratio and *Desulfovibrionaceae* and *Ruminococcaceae* populations in obese rats.

Besides lactic acid bacteria, *Bacillus* species (*Bacillus* spp.) have also been paid attention as potential probiotics, which are useful for treating metabolic disorders [32]. Choi et al. [33] reported that dietary supplementation with *Bacillus licheniformis*-fermented soybean paste prevented obesity and improved glucose metabolism in HFD-fed mice. Their studies also showed that *Bacillus licheniformis* had the effect on the serum lipid levels, especially decreasing the TC level. Similar to previous results, we observed the partial prevention of weight gain and the increasing level of TG induced by HFD after BL treatment. However, we did not find the reshaping of gut microbiota in the BL group compared with the HF group. These results indicate that the potential positive effect of BL on HFD-induced obesity in rat is not based on the modulation of the gut microbiota.

XOS is considered to be novel prebiotics, which has been suggested to improve gut health and stimulate the animals' immune response [34, 35]. XOS consumption has been found to result in increased *Bifidobacterium* populations in human and animal studies [24, 36, 37]. Studies with XOS also indicate the potential to improve the management of glucose metabolism [25, 26]. Thiennimitr et al. [38] reported that XOS treatment attenuated dyslipidemia by HFD as indicated by decreased plasma total and LDL cholesterol levels. In line with the previous study, we have demonstrated that XOS contributed to ameliorate body weight and serum lipid and inflammation parameters in HFD-fed rats (Figures 1 and 2). Moreover, *per se*, XOS was found to have effects on modulating the gut microbiota composition and increasing gut mucosal-protective *Bifidobacteria* levels only in the prebiotic XOS-treated group (Figures 3 and 7), probably due to the bifidogenic effect of XOS [39]. But our results showed that administration of XOS significantly decreased the diversity of the gut microbiota, and this has aroused our attention. In a previous study, Fehlbaum et al. [40] also demonstrated a decrease in alpha diversity with increasing fiber concentration (including XOS), but they found that the total concentrations of SCFAs increased with increasing fiber concentration, which can provide an important energy source for the colon and regulate immune responses of the host. Increasing of SCFAs will lower the pH in gut and may thus affect the growth of some pH-sensitive bacteria such as E. coli [41], resulted in lower bacterial diversity. Also, studies have suggested that lower bacterial diversity may relate to the obesity development [27], but the HFD-induced intestinal dysbiosis may contribute more significantly to this development, especially the unbalanced LPS and SCFA production in the gut. On the other hand, SCFAs, such as butyrate, though a major energy source for colonocytes, has been found to increase mitochondrial activity, prevent metabolic endotoxemia, improve insulin sensitivity, possess anti-inflammatory potential, increase intestinal barrier function, and protect against diet-induced obesity without causing hypophagia [42].

Gut microbiota, particularly *Bacteroidetes* and *Firmicutes*, are two major phyla in human and animal gut microbiota [43, 44], and this phenomenon was also found in our study (Figure 3(a)). Previous studies have suggested that a high ratio of *Firmicutes* to *Bacteroidetes* is often thought to be a key characteristic of obesity [45, 46]. The results presented here showed that HFD-induced rats had a relatively higher F/B ratio compared to the control rats, but these could be inverted by administering XOS or the combination of BL and XOS (Figure 5(b)). Moreover, we found more *Bacteroidetes* in the XOS and XOS-BL groups than in the HF group. *Parabacteroides*, the most abundant in the combination of XOS as well as being identified in DGGE pattern, demonstrated its capacity to alter the bile acid to succinate and secondary bile acids both in vivo and in vitro [47]. They also identified that succinate promotes the intestinal gluconeogenesis (IGN) pathway by activating fructose-1,6-bisphophatase. *Prevotella copri* significantly increases the production of succinate that can be a substrate for IGN. Furthermore, succinate is associated with the inhibition of hepatic glucose production and the improvement of glucose metabolism independently [48]. In parallel, *P. succinatutens* that specialize in the utilization of succinate provided by other bacteria [49] were enriched in the XOS and XOS-BL groups. Furthermore, *Phascolarctobacterium* spp. produces high amount of the short chain fatty acids (SCFAs) [50].

In addition, our results clearly show that *Prevotellaceae*, a subgroup of *Bacteroidetes*, was significantly enriched in the XOS and XOS-BL groups. It was reported that *Prevotellaceae* family was abundant in pectin or whole grain oat, which improved insulin sensitivity and plasma cholesterol profile in previous animal studies [51, 52]. In this study, the increased relative abundance of *Desulfovibrionaceae* was found in HFD-induced obesity rats; however, combination of BL and XOS could significantly reverse the change of this species (*P* < 0.05; Figure 6(a)). Most members of *Desulfovibrionaceae* (*Proteobacteria* phyla) were reported as endotoxins (such as LPS) producers and induce intestinal inflammation, which were thought to be positively associated with obesity [53, 54]. Our result was consistent with the findings in previous research showing that a probiotic-enriched dietary intervention can ameliorate the abundance of *Desulfovibrionaceae* in obese individuals [55]. Interestingly, the relative abundance of *Ruminococcaceae* induced by HFD can be obviously alleviated by combination of BL and XOS treatment (*P* < 0.05; Figure 6(a)). It has been reported that a high abundance of *Ruminococcaceae* was observed in HFD-induced obesity mice [56, 57], and they are strongly linked to obesity [58]. Kieier et al. [59] also verified that *Ruminococcaceae* might negatively affect weight loss rate in dogs by producing acetic and propionic acid. However, some researchers reported that *Ruminococcaceae* was known as butyrate-producing bacteria. Butyrate, a major SCFA, increases insulin sensitivity and energy expenditure in mice [60] and also may have immune modulating effects [61]. As shown in the study by Zhou et al. [62], sodium butyrate attenuates HFD-induced steatohepatitis in mice by strengthening the gut barrier. Endo et al. [63] also verified that butyrate-producing probiotics had beneficial effects in the prevention of NAFLD progression in rats. Therefore, the conclusion remains controversial. More research is required to gain further recognition. Taken together, gut microbiota plays a pivotal role as a modulator of energy homeostasis and fat deposition, and we have reason to believe that the combination of BL and XOS shows an antiobesity effect through beneficial modulation of the gut microbiota.

LPS is the main component of the outer membrane of Gram-negative bacteria, and it is the endotoxin that can induce low-grade inflammation [64, 65]. It was, therefore, postulated that it could be the molecular link between intestinal microflora and the chronic systemic inflammation induced by an HFD. Cani et al. [66] reported that mice fed an HFD for as short term as 2 to 4 weeks exhibited a significant increase in plasma LPS. In our experiments, supplements of combined BL and XOS significantly reduced the serum LPS level (Figure 2(e)). In addition, both the CCA and Spearman's correlation analyses illustrated that the serum and liver levels of LPS were significantly related to microbial variations (Figure 8). For example, *Roseburia*, which is associated with several diseases including irritable bowel syndrome, obesity, type 2 diabetes, nervous system conditions, and allergies [67], showed a positive relationship with serum and liver LPS levels; *Parabacteroides*, which has been reported to reduce the severity of intestinal inflammation in murine models of acute and chronic colitis [68], was negatively correlated with serum and liver LPS levels.

## 5. Conclusion

In conclusion, the present study demonstrated that combined BL and XOS supplementation suppressed the rise of F/B ratio and the elevated abundance of *Desulfovibrionaceae* and *Ruminococcaceae* and LPS levels induced by HFD. These results demonstrated the potential to combine probiotic Zhengchangsheng® and prebiotic xylooligosaccharides as a dietary strategy to ameliorate gut dysbiosis, to improve inflammatory status, and thereby to reduce medical disorders associated with HFD-induced obesity. However, the limitation of this study is that the sample size in the Illumina HiSeq sequencing was not enough; further studies based on a larger number of samples should be conducted to investigate the in-depth mechanisms of how the combined BL and XOS supplementation ameliorates the HFD-induced obesity. Moreover, clinical investigations are warranted to assess whether our promising findings can be translated into a potential way to treat obesity.

## Figures and Tables

**Table 1:** Sequence identities of PCR amplicons derived from DGGE gel.

Band	Strain with highest sequence homology	Identity	Phylum
1	*Lactobacillus gasseri*ATCC 33323 = JCM 1131	95%	*Firmicutes*
2	*Prevotella copri* DSM 18205	97%	*Bacteroidetes*
3	*Lactobacillus animalis*KCTC 3501 = DSM 20602	93%	*Firmicutes*
4	*Prevotella dentalis* DSM 3688	89%	*Bacteroidetes*
5	*Phascolarctobacterium succinatutens* YIT 12067	97%	*Firmicutes*
6	*Bacteroides uniformis* ATCC 8492	96%	*Bacteroidetes*
7	*Bacteroides uniformis* ATCC 8492	97%	*Bacteroidetes*
8	*[Clostridium] saccharolyticum*	96%	*Firmicutes*
9	*Alistipes ihumii* AP11	93%	*Bacteroidetes*
10	*Papillibacter cinnamivorans* DSM 12816	95%	*Firmicutes*
11	*Prevotella copri* DSM 18205	98%	*Bacteroidetes*
12	*Parabacteroides distasonis* ATCC 8503	96%	*Bacteroidetes*
13	*Terrisporobacter glycolicus* ATCC 14880	96%	*Firmicutes*
14	*Muribaculum intestinale*	88%	*Bacteroidetes*
15	*Parabacteroides distasonis* ATCC 8503	96%	*Bacteroidetes*
16	*Stomatobaculum longum*	97%	*Firmicutes*
17	*[Clostridium] saccharolyticum*	89%	*Firmicutes*
18	*Prevotella copri* DSM 18205	93%	*Bacteroidetes*
19	*Marinilabilia salmonicolor* JCM 21150	81%	*Bacteroidetes*
20	*Christensenella massiliensis*	93%	*Firmicutes*
21	*Barnesiella intestinihominis* YIT 11860	82%	*Bacteroidetes*
22	*Muribaculum intestinale*	89%	*Bacteroidetes*
23	*Bifidobacterium pseudolongum PV8-2*	99%	*Actinobacteria*

## Data Availability

All data generated or analyzed during this study are included in this published article or available from the corresponding author upon reasonable request.

## References

[1] Xi B., Liang Y., He T. (2012). Secular trends in the prevalence of general and abdominal obesity among Chinese adults, 1993-2009. *Obesity Reviews*.

[2] Gallus S., Lugo A., Murisic B., Bosetti C., Boffetta P., La Vecchia C. (2015). Overweight and obesity in 16 European countries. *European Journal of Nutrition*.

[3] Baskin M. L., Ard J., Franklin F., Allison D. B. (2005). Prevalence of obesity in the United States. *Obesity Reviews*.

[4] World Health Organization (2000). *Obesity: Preventing and Managing the Global Epidemic*.

[5] Igel L. I., Saunders K. H., Fins J. J. (2018). Why weight? An analytic review of obesity management, diabetes prevention, and cardiovascular risk reduction. *Current Atherosclerosis Reports*.

[6] Sun L., Ma L., Ma Y., Zhang F., Zhao C., Nie Y. (2018). Insights into the role of gut microbiota in obesity: pathogenesis, mechanisms, and therapeutic perspectives. *Protein & Cell*.

[7] Dao M. C., Clément K. (2018). Gut microbiota and obesity: concepts relevant to clinical care. *European Journal of Internal Medicine*.

[8] Bibbò S., Ianiro G., Dore M. P., Simonelli C., Newton E. E., Cammarota G. (2018). Gut microbiota as a driver of inflammation in nonalcoholic fatty liver disease. *Mediators of Inflammation*.

[9] Suez J., Korem T., Zeevi D. (2014). Artificial sweeteners induce glucose intolerance by altering the gut microbiota. *Nature*.

[10] Cani P. D., Amar J., Iglesias M. A. (2007). Metabolic endotoxemia initiates obesity and insulin resistance. *Diabetes*.

[11] de La Serre C. B., Ellis C. L., Lee J., Hartman A. L., Rutledge J. C., Raybould H. E. (2010). Propensity to high-fat diet-induced obesity in rats is associated with changes in the gut microbiota and gut inflammation. *American Journal of Physiology Gastrointestinal and Liver Physiology*.

[12] Hildebrandt M. A., Hoffmann C., Sherrill–Mix S. A. (2009). High-fat diet determines the composition of the murine gut microbiome independently of obesity. *Gastroenterology*.

[13] Guo X., Tang R., Yang S., Lu Y., Luo J., Liu Z. (2018). Rutin and its combination with inulin attenuate gut dysbiosis, the inflammatory status and endoplasmic reticulum stress in paneth cells of obese mice induced by high-fat diet. *Frontiers in Microbiology*.

[14] Markowiak P., Śliżewska K. (2017). Effects of probiotics, prebiotics, and synbiotics on human health. *Nutrients*.

[15] Sonnenburg J. L., Bäckhed F. (2016). Diet-microbiota interactions as moderators of human metabolism. *Nature*.

[16] Food and Agriculture Organization (2006). *Probiotics in Food: Health and Nutritional Properties and Guidelines for Evaluation*.

[17] Arena A., Maugeri T. L., Pavone B., Iannello D., Gugliandolo C., Bisignano G. (2006). Antiviral and immunoregulatory effect of a novel exopolysaccharide from a marine thermotolerant *Bacillus licheniformis*. *International Immunopharmacology*.

[18] Heo J., Kim S. K., Park K. S., Jung H. K., Kwon J. G., Jang B. I. (2014). A double-blind, randomized, active drug comparative, parallel-group, multi-center clinical study to evaluate the safety and efficacy of probiotics (Bacillus licheniformis, Zhengchangsheng® capsule) in patients with diarrhea. *Intestinal Research*.

[19] Du S. X., Jia Y. R., Ren S. Q. (2018). The protective effects of _Bacillus licheniformis_ preparation on gastrointestinal disorders and inflammation induced by radiotherapy in pediatric patients with central nervous system tumor. *Advances in Medical Sciences*.

[20] Li Y., Liu M., Zhou J. (2019). *Bacillus* licheniformisZhengchangsheng® attenuates DSS-induced colitis and modulates the gut microbiota in mice. *Beneficial Microbes*.

[21] Pineiro M., Asp N. G., Reid G. (2008). FAO technical meeting on prebiotics. *Journal of Clinical Gastroenterology*.

[22] Santos A., San Mauro M., Díaz D. M. (2006). Prebiotics and their long-term influence on the microbial populations of the mouse bowel. *Food Microbiology*.

[23] Yang J., Summanen P. H., Henning S. M. (2015). Xylooligosaccharide supplementation alters gut bacteria in both healthy and prediabetic adults: a pilot study. *Frontiers in Physiology*.

[24] Sheu W. H., Lee I. T., Chen W., Chan Y. C. (2008). Effects of xylooligosaccharides in type 2 diabetes mellitus. *Journal of Nutritional Science and Vitaminology*.

[25] Joossens M., Huys G., Cnockaert M. (2011). Dysbiosis of the faecal microbiota in patients with Crohn’s disease and their unaffected relatives. *Gut*.

[26] Deng Y., Li M., Mei L. (2018). Manipulation of intestinal dysbiosis by a bacterial mixture ameliorates loperamide-induced constipation in rats. *Beneficial Microbes*.

[27] Turnbaugh P. J., Hamady M., Yatsunenko T. (2009). A core gut microbiome in obese and lean twins. *Nature*.

[28] Koruda M. J., Rolandelli R. H., Bliss D. Z., Hastings J., Rombeau J. L., Settle R. G. (1990). Parenteral nutrition supplemented with short-chain fatty acids: effect on the smallbowel mucosa in normal rats. *The American Journal of Clinical Nutrition*.

[29] Sakamoto M., Benno Y. (2006). Reclassification of bacteroides distasonis, bacteroides goldsteinii and bacteroides merdae as parabacteroides distasonis gen. nov. comb. nov. parabacteroides goldsteinii comb. nov. and parabacteroides merdae comb. nov. *International Journal of Systematic and Evolutionary Microbiology*.

[30] Park S. K., Kim M. S., Roh S. W., Bae J. W. (2012). Blautia stercoris sp. nov., isolated from human faeces. *International Journal of Systematic and Evolutionary Microbiology*.

[31] Delzenne N. M., Neyrinck A. M., Backhed F., Cani P. D. (2011). Targeting gut microbiota in obesity: effects of prebiotics and probiotics. *Nature Reviews Endocrinology*.

[32] Elshaghabee F. M. F., Rokana N., Gulhane R. D., Sharma C., Panwar H. (2017). Bacillus as potential probiotics: status, concerns, and future perspectives. *Frontiers in Microbiology*.

[33] Choi J.-H., Pichiah P. B. T., Kim M.-J., Cha Y.-S. (2016). Cheonggukjang, a soybean paste fermented with B. licheniformis-67 prevents weight gain and improves glycemic control in high fat diet induced obese mice. *Journal of Clinical Biochemistry and Nutrition*.

[34] Lomax A. R., Calder P. C. (2009). Prebiotics, immune function, infection and inflammation: a review of the evidence. *The British Journal of Nutrition*.

[35] De Maesschalck C., Eeckhaut V., Maertens L. (2015). Effects of xylo-oligosaccharides on broiler chicken performance and microbiota. *Applied and Environmental Microbiology*.

[36] Tateyama I., Hashii K., Johno I. (2005). Effect of Xylooligosaccharide intake on severe constipation in pregnant Women. *Journal of Nutritional Science and Vitaminology (Tokyo)*.

[37] Hsu C. K., Liao J. W., Chung Y. C., Hsieh C. P., Chan Y. C. (2004). Xylooligosaccharides and fructooligosaccharides affect the intestinal microbiota and precancerous colonic lesion development in rats. *The Journal of Nutrition*.

[38] Thiennimitr P., Yasom S., Tunapong W. (2018). Lactobacillus paracasei HII01, xylooligosaccharides, and synbiotics reduce gut disturbance in obese rats. *Nutrition*.

[39] Finegold S. M., Li Z., Summanen P. H. (2014). Xylooligosaccharide increases bifidobacteria but not lactobacilli in human gut microbiota. *Food & Function*.

[40] Fehlbaum S., Prudence K., Kieboom J. (2018). In vitro fermentation of selected prebiotics and their effects on the composition and activity of the adult gut microbiota. *International Journal of Molecular Sciences*.

[41] Duncan S. H., Louis P., Thomson J. M., Flint H. J. (2009). The role of pH in determining the species composition of the human colonic microbiota. *Environmental Microbiology*.

[42] Chakraborti C. K. (2015). New-found link between microbiota and obesity. *World Journal of Gastrointestinal Pathophysiology*.

[43] Eckburg P. B., Bik E. M., Bernstein C. N. (2005). Diversity of the human intestinal microbial flora. *Science*.

[44] Turnbaugh P. J., Ley R. E., Mahowald M. A., Magrini V., Mardis E. R., Gordon J. I. (2006). An obesity-associated gut microbiome with increased capacity for energy harvest. *Nature*.

[45] Ley R. E., Bäckhed F., Turnbaugh P., Lozupone C. A., Knight R. D., Gordon J. I. (2005). Obesity alters gut microbial ecology. *Proceedings of the National Academy of Sciences*.

[46] Anhê F. F., Nachbar R. T., Varin T. V. (2017). A polyphenol-rich cranberry extract reverses insulin resistance and hepatic steatosis independently of body weight loss. *Molecular Metabolism*.

[47] Wang K., Liao M., Zhou N. (2019). *Parabacteroides distasonis* alleviates obesity and metabolic dysfunctions via production of succinate and secondary bile acids. *Cell Reports*.

[48] De Vadder F., Kovatcheva-Datchary P., Zitoun C., Duchampt A., Bäckhed F., Mithieux G. (2016). Microbiota-produced succinate improves glucose homeostasis via intestinal gluconeogenesis. *Cell Metabolism*.

[49] Watanabe Y., Nagai F., Morotomi M. (2012). Characterization of Phascolarctobacterium succinatutens sp. nov. an asaccharolytic, succinate-utilizing bacterium isolated from human feces. *Applied and Environmental Microbiology*.

[50] Duncan S. H., Holtrop G., Lobley G. E., Calder A. G., Stewart C. S., Flint H. J. (2004). Contribution of acetate to butyrate formation by human faecal bacteria. *The British Journal of Nutrition*.

[51] Ivarsson E., Roos S., Liu H. Y., Lindberg J. E. (2014). Fermentable non-starch polysaccharides increases the abundance of Bacteroides-Prevotella-Porphyromonas in ileal microbial community of growing pigs. *Animal*.

[52] Zhou A. L., Hergert N., Rompato G., Lefevre M. (2015). Whole grain oats improve insulin sensitivity and plasma cholesterol profile and modify gut microbiota composition in C57BL/6J mice. *The Journal of Nutrition*.

[53] Delzenne N. M., Cani P. D. (2011). Interaction between obesity and the gut microbiota: relevance in nutrition. *Annual Review of Nutrition*.

[54] Zhang-Sun W., Augusto L. A., Zhao L., Caroff M. (2015). Desulfovibrio desulfuricans isolates from the gut of a single individual: structural and biological lipid A characterization. *FEBS Letters*.

[55] Xiao S., Fei N., Pang X. (2014). A gut microbiota-targeted dietary intervention for amelioration of chronic inflammation underlying metabolic syndrome. *FEMS Microbiology Ecology*.

[56] Wang Z., Lam K. L., Hu J. (2019). Chlorogenic acid alleviates obesity and modulates gut microbiota in high-fat-fed mice. *Food Science & Nutrition*.

[57] Liu Z., Wang N., Ma Y., Wen D. (2019). Hydroxytyrosol improves obesity and insulin resistance by modulating gut microbiota in high-fat diet-induced obese mice. *Frontiers in Microbiology*.

[58] Chávez-Carbajal A., Nirmalkar K., Pérez-Lizaur A. (2019). Gut microbiota and predicted metabolic pathways in a sample of Mexican women affected by obesity and obesity plus metabolic syndrome. *International Journal of Molecular Sciences*.

[59] Kieler I. N., Shamzir Kamal S., Vitger A. D., Nielsen D. S., Lauridsen C., Bjornvad C. R. (2017). Gut microbiota composition may relate to weight loss rate in obese pet dogs. *Veterinary Medicine and Science*.

[60] Gao Z., Yin J., Zhang J. (2009). Butyrate improves insulin sensitivity and increases energy expenditure in mice. *Diabetes*.

[61] Zimmerman M. A., Singh N., Martin P. M. (2012). Butyrate suppresses colonic inflammation through HDAC1-dependent Fas upregulation and Fas-mediated apoptosis of T cells. *American Journal of Physiology Gastrointestinal and Liver Physiology*.

[62] Zhou D., Pan Q., Xin F. Z. (2017). Sodium butyrate attenuates high-fat diet-induced steatohepatitis in mice by improving gut microbiota and gastrointestinal barrier. *World Journal of Gastroenterology*.

[63] Endo H., Niioka M., Kobayashi N., Tanaka M., Watanabe T. (2013). Butyrate-producing probiotics reduce nonalcoholic fatty liver disease progression in rats: new insight into the probiotics for the gut-liver axis. *PLoS One*.

[64] Beutler B., Rietschel E. T. (2003). Innate immune sensing and its roots: the story of endotoxin. *Nature Reviews Immunology*.

[65] Laugerette F., Vors C., Géloën A. (2011). Emulsified lipids increase endotoxemia: possible role in early postprandial low-grade inflammation. *The Journal of Nutritional Biochemistry*.

[66] Cani P., Delzenne N. M., Tuohy K. (2009). The role of the gut microbiota in energy metabolism and metabolic disease. *Current Pharmaceutical Design*.

[67] Tamanai-Shacoori Z., Smida I., Bousarghin L. (2017). Roseburiaspp.: a marker of health?. *Future Microbiology*.

[68] Kverka M., Zakostelska Z., Klimesova K. (2011). Oral administration of Parabacteroides distasonis antigens attenuates experimental murine colitis through modulation of immunity and microbiota composition. *Clinical and Experimental Immunology*.

